# Diagnosing sarcopenia with semi-automated skeletal muscle computed tomography cutoff values and the association of these muscle metrics with long-term physical exercise

**DOI:** 10.1016/j.redii.2023.100026

**Published:** 2023-03-30

**Authors:** Robert Janiszewski, Nathan Law, Ryan Walters, Tami DenOtter

**Affiliations:** a Creighton University School of Medicine, 2500 California Plaza, Omaha, NE 68178, 68124, USA; b Creighton University School of Medicine, Department of Clinical Research and Public Health, 2500 California Plaza, Omaha, NE 68178, USA; c Creighton University School of Medicine, Department of Radiology, 7500 Mercy Rd, Omaha, NE 68124, USA

## 1 Introduction

The increased life expectancy in developed countries has led to an increase in degenerative diseases in the aging population. Sarcopenia, an age-related degenerative loss of muscle mass and muscle function, is an independent risk factor for various adverse outcomes including falls and fractures [1], loss of independence and hospitalization [2], major postoperative complications [3], and death [4,5]. Sarcopenia is associated with a major increase in morbidity and mortality in the elderly population, and it is also associated with increased healthcare costs [6]. This has directly led to an increase in public interest and research in the area of sarcopenia. In 2018, the International Clinical Practice Guidelines for Sarcopenia (ICFSR) were established for the screening, diagnosis, and management of sarcopenia. The guidelines include the screening of individuals aged 65 or older, or after the occurrence of a major health event. Guidelines state screening should be done opportunistically [7].

Most sources agree that the diagnosis of sarcopenia should include both muscle mass and muscle function [8,9]. Muscle mass has typically been evaluated by imaging, whereas muscle function has previously been largely limited to the more time-intensive measurement of patient muscle strength (e.g., grip strength) or physical performance (e.g., gait speed). Recent advancements in Quantitative Computed Tomography (QCT) imaging have resulted in this modality being considered the gold standard in evaluating muscle. QCT can measure both muscle mass and muscle quality as it relates to fatty infiltration. A single axial Computed Tomography (CT) image can be used to measure Skeletal Muscle Area (SMA) (cm^2^) and muscle quality via Skeletal Muscle Radiation Attenuation (SMRA), measured via Hounsfield Units (HU), as a precise and valid diagnostic tool [10]. Cross-sectional SMA at the third lumbar (L3) vertebra, where skeletal muscle peaks, is highly correlated to total skeletal muscle [11]. Adjusting SMA for height squared yields Skeletal Mass Index (SMI) (cm^2^/m^2^), a measurement of relative muscle mass [12]. SMRA is a measurement of muscle quality that is inversely related to muscle fat content and has been associated with physical function [13]. CT may provide the necessary tools to opportunistically evaluate for sarcopenia via assessment of these muscle metrics. Opportunistic CT imaging implies gathering additional data from the CT when the CT is obtained for other diagnostic purposes without the addition of radiation or cost to the patient. Currently, the evaluation of SMA, SMI, and SMRA on CT is limited since semi-automated post-processing of the CT data is needed at most institutions to compute these muscle metrics clinically. However, advancements in artificial intelligence to evaluate muscle metrics in a fully automated process are occurring rapidly. A fully automated process of measuring these metrics will likely advance this technology from the research setting into clinical practice [14]. Automated techniques for opportunistic evaluation of muscle metrics show promise in providing a standardized, reproducible, accessible, and cost-effective method for assessing sarcopenia indices, thus creating greater potential for disease recognition and clinical intervention [15]. The much-added value of obtaining muscle metrics opportunistically may help increase both physicians' and patients’ awareness of this silent condition with earlier detection, consequently reducing the increased morbidity, mortality, and cost associated with this age-related decrease in muscle mass and quality [16].

The European Working Group on Sarcopenia in Older People (EWGSOP) established guidelines in 2018 to define sarcopenia. Based on these guidelines, the potentiality for a sarcopenia diagnosis exists in individuals found to have muscle weakness. Once muscle weakness is identified through various testing methods, sarcopenia can then be confirmed by identifying deficits in either muscle quantity or muscle quality [17]. Additionally, EWGSOP has given a consensus recommendation for the diagnosis of sarcopenia to be defined with sex-specific cutoff values two standard deviations below the mean of a healthy, young adult population. Sex-specific QCT cutoff values of SMA and SMI were established using a healthy US population at L3. SMA cutoff values for males and females at the L3 level are 144.3 cm^2^ and 92.2 cm^2^, respectively. SMI cutoff for males and females at the L3 level is 45.4 cm^2^/m^2^ and 34.4 cm^2^/m^2^ respectively. These cutoff values have been supported and validated in later research [18,19]. SMRA cutoff values at the level of L3 for males and females are 38.5 HU and 34.3 HU respectively. In the absence of physical function testing, SMRA can be used as a surrogate to assess muscle function [19].

The association between these CT-derived muscle metrics and adverse patient outcomes has been well studied, especially in cancer patients, trauma patients, and patients undergoing surgery. Although much work has been done in establishing the diagnostic and prognostic value of these CT measurements as used to define sarcopenia, few studies have looked at the prevention of sarcopenia as it relates to these metrics. Whereas prior studies related to the prevention of sarcopenia have largely examined the effects of exercise and protein intake in the prevention of sarcopenia as related to muscle function as measured with patient's muscle strength (e.g., grip strength) or physical performance (e.g., gait speed), the goal of this study was to identify trends in the QCT muscle metrics of SMA, SMI, and SMRA with respect to varying degrees of physical exercise. Additionally, this study aimed to determine if physical exercise lowered the odds of developing sarcopenia as defined by the newly derived diagnostic skeletal muscle cutoff values. If trends can be appropriately identified, screening and preventative measures may eventually be developed for at-risk populations to reduce the progression to sarcopenia, thus decreasing the associated burden of morbidity, mortality, and healthcare costs.

## 2 Materials and methods

### 2.1 Study population and participants

This research study was performed as a cross-sectional prospective study at a tertiary medical center. Eligible study patients included those who were scheduled for a routine outpatient CT exam to include the abdomen and pelvis, regardless of the indication for the CT. Both men and women ages 60–89 years were eligible for the study. Institutional Review Board (IRB) approval was granted by the Creighton University IRB Board (2,000,592).

Eligible study patients meeting inclusion criteria were first identified by a researcher clinical coordinator. Informed consent was obtained by the clinical coordinators. The eligible study patients were asked to answer a one-page questionnaire addressing physical activity, smoking history, and cancer status. Their height and weight were obtained from the patients’ medical charts. Demographic data including gender, age, and ethnicity were also obtained by the clinical coordinators. Patients under the age of 60 were excluded from the study given their low likelihood of meeting the skeletal muscle cutoff values for sarcopenia diagnosis.

### 2.2 CT scan protocol

CT scans were obtained on patients who presented to our medical center for a diagnostic CT of the abdomen and pelvis ordered for various clinical indications, met inclusion criteria and consented to take part in this study. CT imaging followed our Radiology Departmental routine CT imaging protocol using Care Dose modulation, to include helical mode scanning in feet-first position with 120 kV with a gantry rotation of 0.5 s and without gantry tilt. CT imaging was performed on a Siemens Definition Dual Source 64 slice-MDCT scanner (Siemens Healthcare, Munich, Germany) in the outpatient setting.

On contrast-enhanced CT examinations, 100 ML of Isovue 370 (Bracco Diagnostics, Milan, Italy) was administered at 2 ml/second. The portal venous phase of imaging with a 65-second delay was used for muscle measurements when contrast was administered.

### 2.3 Skeletal muscle analyses/measurements

**Fig. 1:**
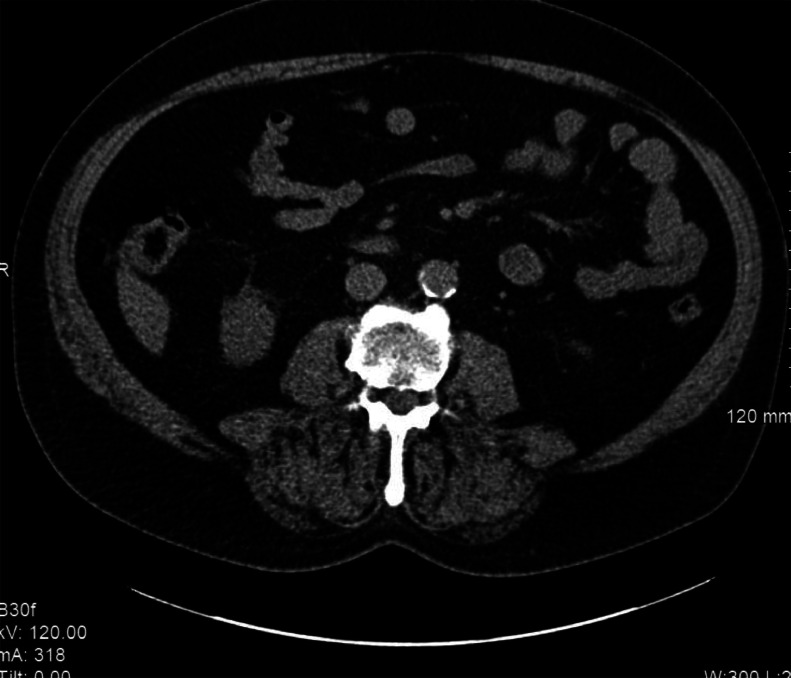
Axial Image of 80-year-old male at inferior aspect of L3.

**Fig. 2:**
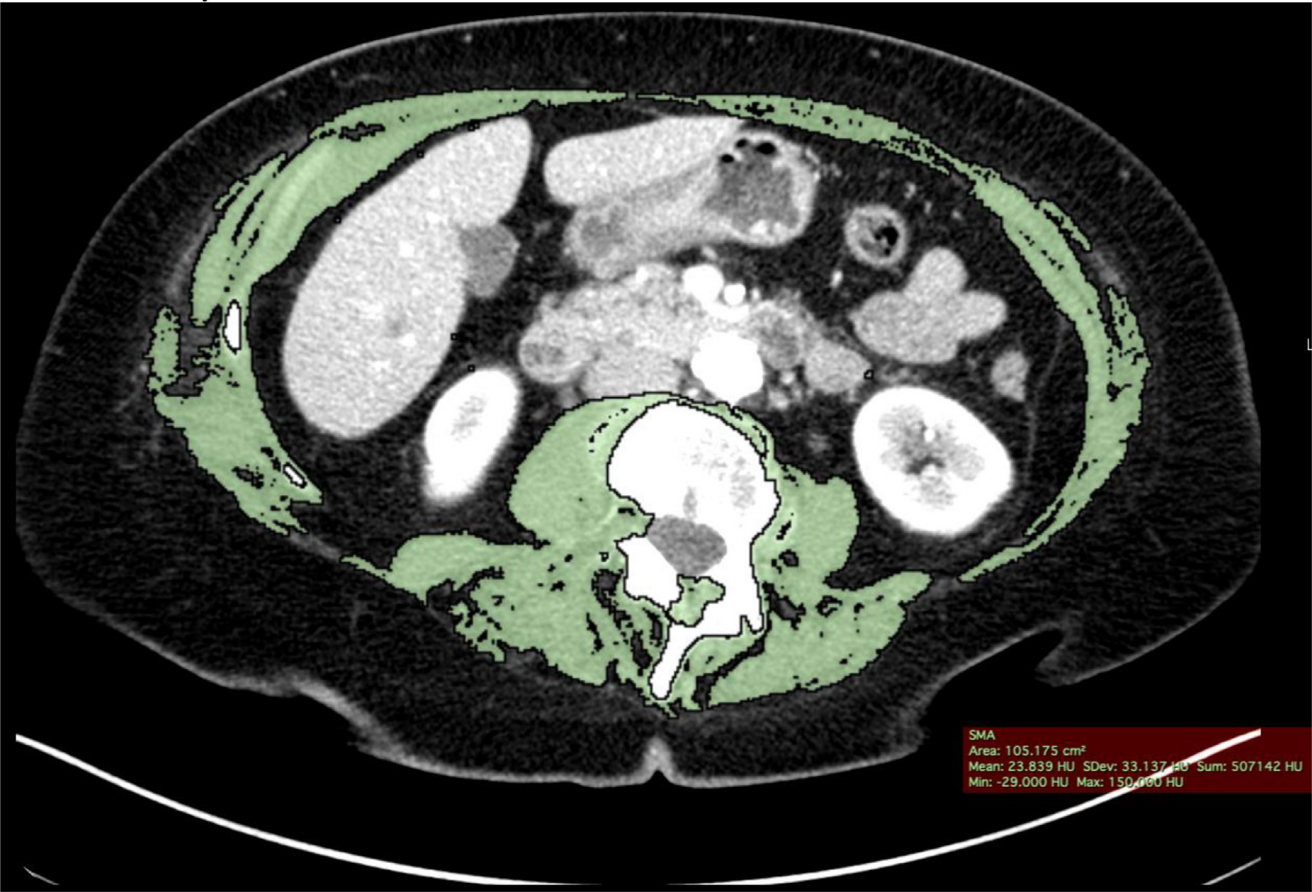
In Color - Axial image of 76-year-old female at inferior aspect of L3 with Region of Interest Overlay.

Skeletal muscle measurements were obtained opportunistically on the CTs of the study patients by post-process semi-automated algorithms to assess skeletal muscle. SMA was measured on a single axial slice nearest the inferior aspect of the L3 vertebral body as the area of pixels within −29 to +150 HU to define muscle tissue, as previously validated [[18], [19], [20], [21]]. The L3 region contains psoas, paraspinal muscles (erector spinae, quadratus lumborum), and abdominal wall muscles (transversus abdominus, external and internal obliques, rectus abdominus). The Aycan OsiriX Pro software (Pixmeo, Geneva, Switzerland) was used to compute SMA by summing the cross-sectional muscle area on this single axial CT slice and multiplying by pixel surface area. SMI was calculated by correcting SMA for the patient's height. SMRA was also computed with the Aycan OsiriX Pro software program on the same single axial slice nearest the inferior aspect of the L3 vertebral body as the area of pixels within −29 to +150 HU to define muscle tissue. SMRA was determined by the software application by averaging the attenuation (HU) for the selected pixels within the defined area of muscle. These semi-automated measurements were obtained by 3 different researchers: one faculty musculoskeletal radiologist with 14 years of practice experience after fellowship training and 2 medical students after detailed training and direct supervision by the faculty musculoskeletal radiologist. Fig. 1, Fig. 2 demonstrate the technique used to identify muscle metrics.

### 2.4 Physical activity measurements

A One-Page Questionnaire on Long-Term Recreational Physical Activity was used to assess physical activity; a validated physical activity questionnaire that was used for the VITamins And Lifestyle (VITAL) Study [22].

The questionnaire evaluated recreational physical activities (walking exercise, weightlifting, yoga), mild exercise, and moderate-strenuous exercise with calculations of Met-hours/week for each activity. A total Met-hours/week was then calculated by summing all three different physical activity categories (recreational, mild exercise, and moderate-strenuous exercise). The questionnaire was given to the consented patients at the time of CT imaging and all data was calculated in a similar fashion as the validated VITamins And Lifestyle (VITAL) study.

Three recreational physical activities were specifically assessed (walking, weightlifting, and yoga) and two additional categories of mild exercise (including golf, dancing, or bowling) or moderate-strenuous exercise (including light conditioning, water aerobics, aerobics class, running/jogging, aerobics, folk dancing, swimming, cycling, stair machine, tennis, racquetball, squash, or other). The patients were instructed to only report activities done regularly, at least once per week for at least one year during the past ten years. All responses were categorical. For each activity, the patients were asked to indicate the duration of exercise (average minutes/day), frequency (days/week), and the number of years in the past 10 years. For walking they were asked to report their usual pace; casual walking pace (each mile takes 30 min or more), moderate pace (each mile takes 20 −29 min), or fast pace (each mile takes 19 min or less). For the moderate-strenuous exercise (including light conditioning, water aerobics, aerobics class, running/jogging, aerobics, folk dancing, swimming, cycling, stair machine, tennis, racquetball, squash or other) patients were asked to select one or two of the exercises done most often over the past 10 years.

Similar to Littman et al., for each activity, an intensity code (MET) was assigned to that activity and MET-hour per week was calculated. The MET assigned to each physical activity was based on a compendium of physical activity that is commonly used to quantify the energy costs of various activities [22]. MET-hours were calculated independent of patient body size. MET-hour per week calculated for each activity over the preceding 10 years and calculated as follows:

[frequency of activity per week x minutes per session x years in the past 10 x MET for activity] divided by [(60 min/hour) x 10 years]

The sum of the MET hours/week of each activity was then added together to represent the total MET hours/week.

Similar to Littman et al., assumptions of the categorical data were made to be the midpoint of the range indicted by the patient in the questionnaire [22]. Frequency, minutes, and years were all assumed to be the mid portion of the range selected by the patient.

### 2.5 Statistical analysis

Depending on data distribution, continuous variables are presented as mean and standard deviation or median and interquartile range; the minimum and maximum are always provided. Categorical variables are presented as count and percent. Pearson correlations were estimated between the three skeletal muscle indicators of SMA, SMI, and SMRA. Separate unadjusted linear regression models were estimated for each skeletal muscle indicator with physical activity in METs serving as the primary independent variable and age, biological sex, BMI, smoking status, and history of cancer serving as covariates. Adjusted models were estimated to include METs as well as any statistically significant covariate from the unadjusted model. A diagnosis of sarcopenia was determined separately for each skeletal muscle indicator using the indicator-specific threshold. Separate unadjusted logistic regression models were estimated to quantify the odds of sarcopenia diagnosis across physical activity in METs. For all regression models, the functional form for all continuous covariates was estimated using restricted cubic splines with pre-specified knot points at the 10th, 50th, and 90th percentiles; the decision to retain a non-linear functional form was dictated by likelihood ratio test when compared to a model estimated with linear functional form. SAS v. 9.4 was used for all statistical analysis with two-tailed *p* < .05 used to indicate statistical significance.

## 3 Results

### 3.1 Study population/participants

Ninety-five patients enrolled in the study, of whom two patients withdrew from the study, two patients were excluded from the study due to technical issues from their CT imaging which led to non-diagnostic CT data, and two patients were excluded from the study due to an incomplete physical activity questionnaire. These exclusions resulted in final data for 89 study patients. Of the 89 patients evaluated in this study, 40 (44.9%) were female, 49 (55.1%) were male, and 87 (97.8%) identified as White. The average age was 69.8 (SD: 6.8 years, range: 60–89 years) and the average BMI was 29.4 (SD: 7.0, range: 19.0–57.8). Finally, 13 (14.6%) were current smokers, 39 (43.8%) were former smokers, and 37 (41.6%) never smoked; 41 (46.1%) patients had a history of cancer.

### 3.2 Skeletal muscle analysis and sarcopenia diagnostic cutoff values

SMA, SMI, and SMRA were measured via QTC. The average SMA was 139.6 (SD: 33.7, range: 74–232) with 18 (20.2%) patients meeting sarcopenia criteria based on SMA cutoff values defined by Derstine et al. [19]. The average SMI was 48.0 (SD: 9.2, range: 28–82) with 16 (18.0%) patients meeting sarcopenia criteria based on SMI cutoff values defined by Derstine et al. The average SMRA was 24.7 (SD: 8.7, range: 6–46) with 80 (89.9%) patients meeting sarcopenia criteria based on SMRA cutoff values defined by Derstine et al. The correlation between SMA and SMI was 0.88 (*p* < 0.001), which was expected given SMA and SMI are effectively transformations of each other. The correlation between SMA and SMRA and between SMI and SMRA were similar (0.26, *p* = 0.014 and 0.24, *p* = 0.026, respectively).

### 3.3 Physical activity

Different exercise modalities surveyed included recreational activity (walking, weightlifting, yoga, mild exertion, and moderate exertion). Seventy-three (82.0%) participants reported engaging in walking as a significant form of exercise, for whom the median number of METs was 4.9 (IQR: 2.0–7.0, range: 0.1–18.0). Seventeen (19.1%) participants reported engaging in weightlifting as a significant form of exercise, for whom the median number of METs was 3.5 (IQR: 0.9–7.1, range: 0.3–21.0). Seven (7.9%) participants reported engaging in yoga as a significant form of exercise, for whom the median number of METs was 1.5 (IQR: 0.3–4.2, range: 0.3–21.0). Thirty-seven (41.6%) participants reported engaging in mild exertion activity, for whom the median number of METs was 2.5 (IQR: 1.1–4.4, range: 0.3–17.5). Finally, 33 (37.1%) participants reported engaging in moderate-strenuous exertion activity as a significant form of exercise, for whom the median number of METs was 7.0 (IQR: 2.2–15.8, range: 0.5–48.0).

### 3.4 SMA and physical activity

**Fig. 3:**
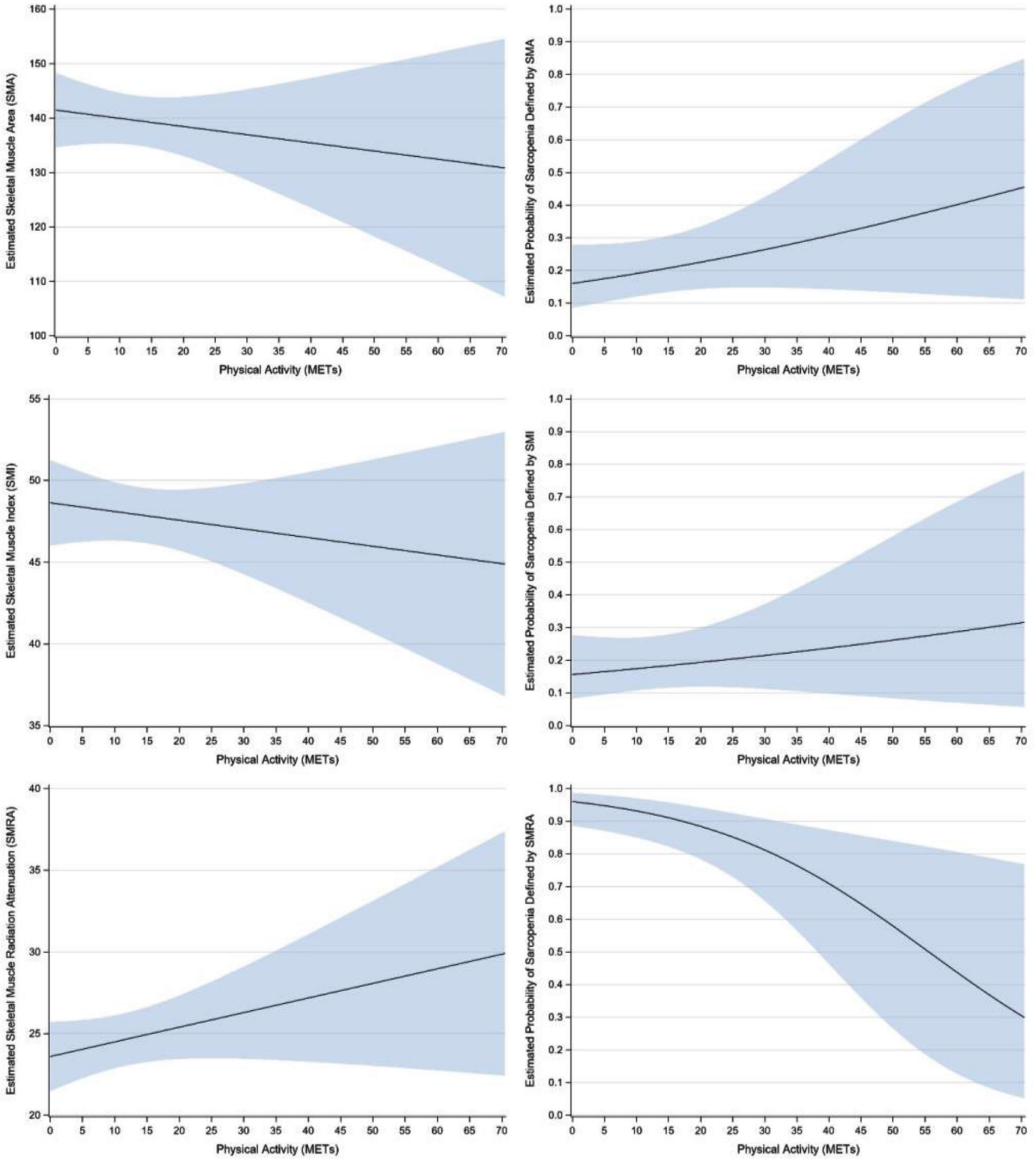
Estimated skeletal muscle indicator and the probability of sarcopenia diagnosis using specific muscle indicator cut-offs for SMA, SMI, SMRA.

Physical activity was not associated with SMA. In the adjusted model, one-MET higher was associated with an average of 0.2-units lower SMA (95% CI: 0.6-units lower to 0.3-units higher, *p* = .460; [Fig. 3 and appendix Table A.1). SMA was associated with age and biological sex. Being one-year older at study entry was associated with an average of 1.3-units lower SMA (95% CI: −2.0 to −0.6, *p* < .001) and females averaged 49.4-units lower SMA compared to males (95% CI: −58.5 to −10.3, *p* < .001). Further, one-unit higher METs was not associated with the odds of sarcopenia diagnosis (OR: 1.02, 95% CI: 0.99–1.06, *p* = .212; Fig. 3 shows predicted probability of sarcopenia diagnosis).

**Fig. A.1:**
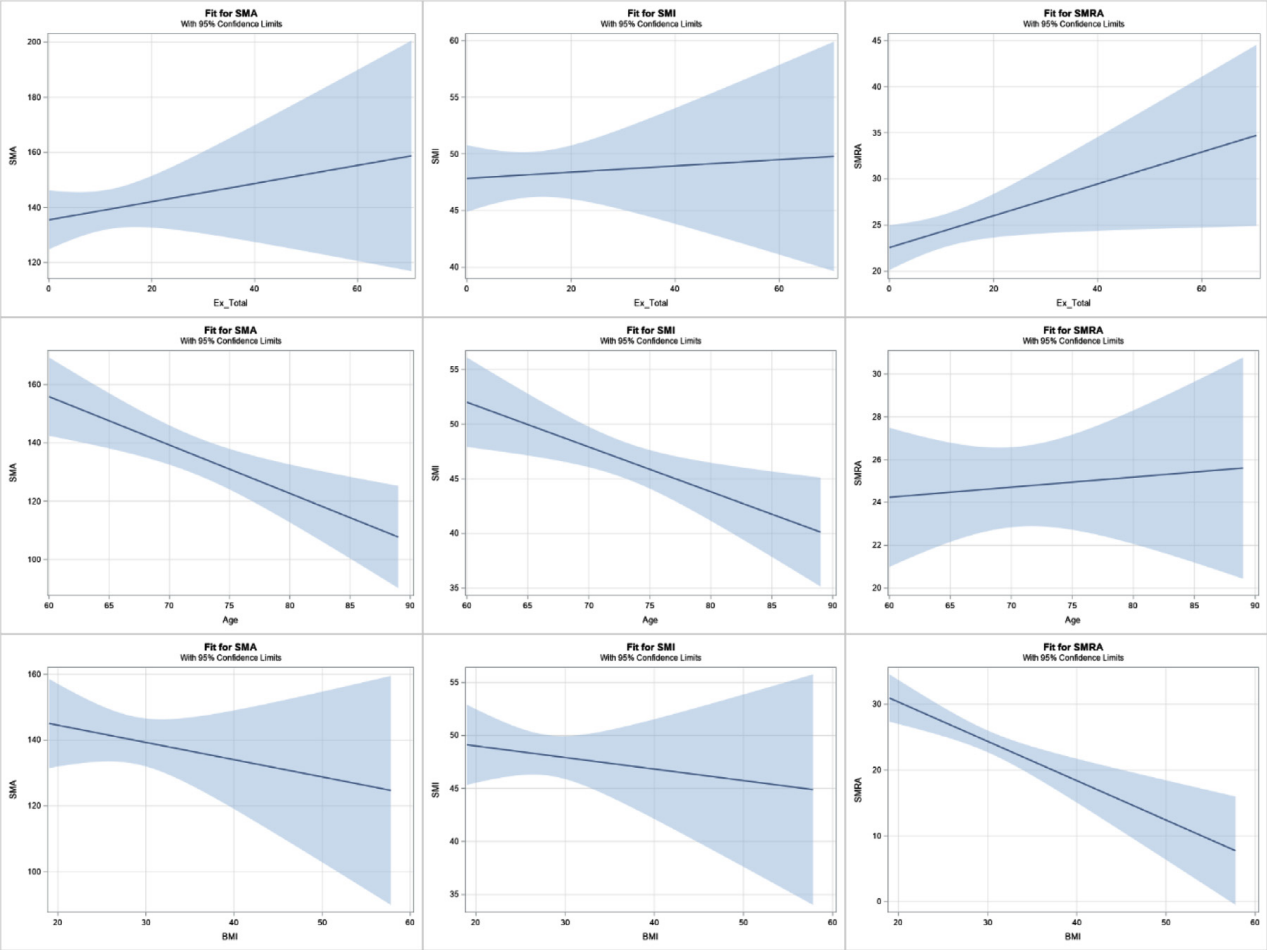
The three columns are defined by skeletal muscle indicator—SMA (first column), SMI (second column), and SMRA (third column). The three rows are unadjusted effects for Total METs (first row), Age (second row), and BMI (third row). In each Figure, the thick blue line shows the predicted skeletal muscle indicator (*y*-axis) at any given level of the predictor variable (*x*-axis); the direction and magnitude of the slope maps directly onto the Slopes provided in Tables A.1, A.2, and A.3. Shaded areas represent 95% confidence intervals.

**Table A.1:** Linear regression results for SMA.

		Unadjusted		Adjusted	
	Descriptive Statistic	Slope (95% CI)	*p*	Slope (95% CI)	*p*
Total METs	Fig. A.1	0.3 (−0.4 to 1.0)	0.351	−0.2 (−0.6 to 0.3)	0.460
Age	Fig. A.1	−1.7 (−2.6 to −0.7)	0.001	−1.3 (−2.0 to −0.6)	<0.001
Biological Sex					
Female	111.9 ± 18.0	−50.3 (−59.6 to −40.9)	<0.001	−49.4 (−58.5 to −10.3)	<0.001
Male	162.2 ± 25.7	Reference		Reference	
BMI	Fig. A.1	−0.5 (−1.7 to 0.7)	0.378		
Smoking Status					
Current	140.3 ± 31.8	5.3 (−16.5 to 27.2)	0.629		
Former	143.7 ± 31.9	8.7 (−7.1 to 24.6)	0.277		
Never	135.0 ± 36.4	Reference			
Cancer Diagnosis					
Yes	135.3 ± 31.7	−7.8 (−22.2 to 6.6)	0.285		
No	143.1 ± 35.6	Reference			

*Note*. For continuous predictors (i.e., Total METs, Age, BMI), the Slope indicates the expected difference per one-unit higher predictor value. For example, one-MET higher was associated with 0.3-unit higher SMA. For binary predictors (i.e., Biological Sex, Smoking Status, Cancer Diagnosis), the Slope represents the difference between one category and the reference category. For example, Females averaged 50.3-units lower SMA compared to males. Only predictors that were statistically significant in the unadjusted model were included as covariates in the adjusted model.

### 3.5 SMI and physical activity

Physical activity was not associated with SMI. In the adjusted model, one-MET higher was associated with an average of 0.1-units lower SMI (95% CI: 0.2-units lower to 0.1-units higher, *p* = .455; Fig. 3 and appendix Table A.2). SMI was associated with age and biological sex. Being one-year older at study entry was associated an average of 0.3-units lower SMI (95% CI: −0.6 to −0.1, *p* = .014) and females averaged 8.5-units lower SMI compared to males (95% CI: −12.0 to −5.0, *p* < .001). One-unit higher METs was not associated with the odds of sarcopenia diagnosis (OR: 1.01, 95% CI: 0.98–1.05, *p* = .496; Fig. 3).

**Table A.2:** Linear regression results for SMI.

		Unadjusted		Adjusted	
	Descriptive Statistic	Slope (95% CI)	*p*	Slope (95% CI)	*p*
Total METs	Fig. A.1	0.0 (−0.1 to 0.2)	0.688	−0.1 (−0.2 to 0.1)	0.455
Age	Fig. A.1	−0.4 (−0.7 to −0.1)	0.006	−0.3 (−0.6 to −0.1)	0.014
Biological Sex					
Female	43.2 ± 6.7	−8.6 (−12.0 to −5.3)	<0.001	−8.5 (−12.0 to −5.0)	<0.001
Male	51.9 ± 9.1	Reference		Reference	
BMI	Fig. A.1	−0.1 (−0.5 to 0.3)	0.550		
Smoking Status					
Current	49.5 ± 7.5	2.5 (−3.0 to 8.0)	0.370		
Former	48.4 ± 8.5	1.5 (−2.9 to 5.9)	0.508		
Never	47.0 ± 10.4	Reference			
Cancer Diagnosis					
Yes	46.8 ± 8.3	−2.2 (−6.0 to 1.7)	0.267		
No	49.0 ± 9.8	Reference			

*Note*. For continuous predictors (i.e., Total METs, Age, BMI), the Slope indicates the expected difference per one-unit higher predictor value. For example, being one-year older was associated with 0.4-unit lower SMI. For binary predictors (i.e., Biological Sex, Smoking Status, Cancer Diagnosis), the Slope represents the difference between one category and the reference category. For example, Current smokers averaged 2.5-units higher SMI compared to Never smokers. Only predictors that were statistically significant in the unadjusted model were included as covariates in the adjusted model.

### 3.6 SMRA and physical activity

In the unadjusted model, physical activity was associated with SMRA with one-unit higher METs associated with 0.2-unit higher SMRA (95% CI: 0.0 to 0.3, *p* = .036); however, this association was not significant in the adjusted model (0.1, 95% CI: 0.0 to 0.2, *p* = .153; Fig. 3 and appendix Table A.3). SMRA was associated with BMI and history of cancer. Specifically, one-unit higher BMI was associated with a 0.5-unit decrease in SMRA (95% CI: −0.8 to −0.2, *p* = .004) and patients with a history of cancer averaged 3.4-units lower SMRA compared to participants without a history of cancer (95% CI: −6.6 to −0.1, *p* = .045). Finally, one-unit higher METs was associated with 6% lower odds of sarcopenia as defined by SMRA (95% CI: 2% to 9%, *p* = .005; Fig. 3).

**Table A.3:** Linear regression results for SMRA.

		Unadjusted		Adjusted	
	Descriptive Statistic	Slope (95% CI)	*p*	Slope (95% CI)	*p*
Total METs	Fig. A.1	0.2 (0.0 to 0.3)	0.036	0.1 (0.0 to 0.2)	0.153
Age	Fig. A.1	0.0 (−0.2 to 0.3)	0.718		
Biological Sex					
Female	21.9 ± 8.2	−5.2 (−8.8 to −1.6)	0.005	−2.7 (−6.0 to 0.6)	0.112
Male	27.0 ± 8.6	Reference		Reference	
BMI	Fig. A.1	−0.6 (−0.9 to −0.3)	<0.001	−0.5 (−0.8 to −0.2)	0.004
Smoking Status					
Current	27.6 ± 9.7	2.4 (−4.0 to 8.9)	0.452		
Former	23.3 ± 7.4	−1.9 (−5.9 to 2.1)	0.346		
Never	25.2 ± 9.6	Reference			
Cancer Diagnosis					
Yes	22.6 ± 7.5	−3.9 (−7.5 to −0.3)	0.033	−3.4 (−6.6 to −0.1)	0.045
No	26.5 ± 9.4	Reference		Reference	

*Note*. For continuous predictors (i.e., Total METs, Age, BMI), the Slope indicates the expected difference per one-unit higher predictor value. For example, one-MET higher was associated with 0.2-unit higher SMRA. For binary predictors (i.e., Biological Sex, Smoking Status, Cancer Diagnosis), the Slope represents the difference between one category and the reference category. For example, patients with a Cancer Diagnosis averaged 3.9-units lower SMRA compared to patients without a Cancer Diagnosis. Only predictors that were statistically significant in the unadjusted model were included as covariates in the adjusted model.

## 4 Discussion

### 4.1 Significance of the findings

Age was found to have a negative association with both SMA and SMI, the two variables used to assess muscle quantity. The adjusted linear regression for age and SMA had a slope of −1.3 (*P* < .001) and the adjusted linear regression for age and SMI had a slope of −0.3 (*p* < .014). In other words, each additional year of age was associated with a 1.3-unit decrease in SMA and a 0.3-unit decrease in SMI. This demonstrates the correlation between age and decreasing skeletal muscle, illustrating how these muscle metrics are useful in assessing the susceptibility that the aging populations has to decreasing muscle. In agreement with trends that have been proven in previous studies, female biological sex was associated with significantly decreased levels of SMA and SMI relative to their male counterparts. This reduced SMA and SMI in female participants is expected and is accounted for in the lower diagnostic cutoff values for both SMA and SMI in females. These correlations highlight at-risk populations that would most benefit from well-defined screening criteria for sarcopenia and interventions aimed at reducing progression to sarcopenia. These findings concur with the current guidelines of screening of individuals aged 65 or older [7].

BMI and SMRA, the variable used to assess muscle quality, were found to be negatively correlated. The adjusted linear regression for BMI and SMRA resulted in a slope of −0.5 (*p* < .004). This data suggests that each additional unit increase in BMI corresponded with a 0.5 decrease in SMRA. This correlation is predictable as SMRA is a metric for muscle quality; higher BMI can result in higher levels of myosteatosis, consequently reducing SMRA. Not only does myosteatosis have the potential to reduce SMRA levels resulting in an increased incidence of sarcopenia as defined by SMRA, but it may also have the potential to obscure the relevance of SMA and SMI as predictors of sarcopenia in overweight populations as increasing tissue adiposity may result in maintained or even increased muscle surface area [23]. This suggests that the area-based assessment of muscle to evaluate for the muscle-mass component of sarcopenia could potentially lead to underdiagnosis of sarcopenia in overweight or obese populations due to an increasing false negative rate when compared to qualification for sarcopenia based on SMRA, thus inappropriately excluding those with so-called sarcopenic obesity [24]. As the diagnosis of sarcopenia requires both low muscle strength and either low muscle quality as defined by SMRA or low muscle mass as defined by either SMA or SMI, these findings suggest that individuals with a truly low quantity of muscle mass but adequate muscle area due to myosteatosis may be inappropriately deemed as having an adequate area of muscle mass, thus excluding them from consideration for sarcopenia based on muscle quantity metrics.

Unadjusted linear regression showed total METs of exercise and SMRA to be positively correlated. The unadjusted linear regression for total METs and SMRA was identified to have a slope of 0.2 (*p* = .036), meaning that each additional MET was associated with an increase of SMRA of 0.2. The adjusted linear regression of SMRA and total METs was 0.1 (*p* = .153). A correlation between SMRA and exercise is sensible as muscle use should correspond to increasing quality of muscle through diminishing fatty infiltration. Though the significance of the correlation diminished with the adjusted linear regression, it is possible that the results were limited by the number of participants in our study. The temporal coincidence of this study and the COVID-19 pandemic resulted in far lower enrollment than originally anticipated, reducing the power of the study. The identified 0.1 increase in SMRA per MET-hour serves to signal that a more robust, multicenter study could further elucidate a significant correlation between exercise and SMRA. Further elucidating the strength of this correlation may be important in establishing recommended behavioral interventions in at-risk populations to reduce the overall risk of progression to sarcopenia based on muscle quality metrics. This study quantified exercise based on MET-hours, a unit of measurement that attempts to translate a broad range of physical activities into functional standardized units. However, different modalities of exercise may likely induce different results in muscle metrics as defined by SMRA, SMA, and SMI. Further research is needed to categorize the effects that different modalities of exercise have on the various QCT muscle metrics so that the metric that best predicts the morbidity and mortality associated with sarcopenia can be directly targeted via exercise interventions.

In addition to the positive correlation between SMRA and total METs of exercise identified via linear regression, total METs of exercise was associated with lower odds of being diagnosed with sarcopenia using the SMRA threshold. An odds ratio of 0.94 (*p* = .005) between SMRA and total METs of exercise was found (Table 4). This data suggests that one MET-hour increase in exercise in a given patient from our cohort was associated with 6% lower odds of identifying sarcopenia based on SMRA cutoff values. Neither SMA nor SMI was found to have a significant odds ratio with METs of exercise when applying the same statistical method to these variables. This reinforces that exercise can have significant effects on sarcopenia muscle metrics and suggests that trends in SMRA may be the most useful metric for tracking muscle response to exercise. This data further demonstrates the positive association of SMRA and total METs of exercise, reinforcing the need for higher power studies to be performed to better characterize this correlation so that efforts can be made to establish recommendations for diminishing progression toward sarcopenia in at-risk populations.

Based on the newly derived SMA and SMI cutoff values, 20.2% and 18% of participants were identified as meeting the muscle mass cutoff value criteria for sarcopenia respectively. The similarity between SMA and SMI is sensible as SMI is simply a transformation of SMA. In contrast, 89.9% of participants were identified as meeting the muscle quality cutoff values for sarcopenia based on SMRA. This discrepancy highlights the distinct difference between that which is being measured by SMA, SMI, and SMRA, and suggests that isolated use of either metrics for muscle quality (SMRA) or muscle quantity (SMA, SMI) in conjunction with the required muscle weakness that defines sarcopenia may result in an unreliable diagnosis of this disease. As the diagnosis requires both muscle weakness and either low muscle quality or low muscle quantity, the extreme discordance between the likelihood of identifying potential sarcopenia based on muscle quality and quantity metrics is significant. The fact that these muscle metrics are measuring different characteristics of the muscle is illustrated by the axial image in Fig. 1. The patient did not meet cutoff criteria for sarcopenia based on SMA and SMI cutoff values; however, he fell below the SMRA diagnostic cutoff value. The image demonstrates that his muscle area was relatively maintained but muscle attenuation was not maintained due to partial fatty replacement of the muscles.

This inconsistency may be explained by myosteatosis allowing the muscle to maintain normal size but not attenuation. The mean BMI of our cohort was 29.4, which qualifies as overweight and falls just below the cutoff for obesity. Increased BMI is correlated with increased myosteatosis [25]. It is reasonable to predict that adipose invasion of muscle tissue can alter muscle surface area. Thus, it follows that populations with higher BMIs may be underdiagnosed with sarcopenia based on SMA and SMI as these metrics are largely based on muscle surface area. This further highlights a potential deficit in the use of the muscle mass metrics SMA and SMI in assessing for sarcopenia as it suggests that there is increased potential for false negative findings depending on patient BMI. Conversely, it is sensible that a high proportion of this population was found to have sarcopenia based on SMRA cutoffs, likely for similar reasons: SMRA is a measurement of muscle attenuation, therefore high levels of myosteatosis in an overweight-obese population could result in a large number of these individuals having decreased muscle quality as defined by SMRA. This potential trend for an increased association of sarcopenia with BMI must be considered as other literature has suggested that low BMI is typically predictive of increased incidence of sarcopenia [26]. Contradicting evidence has suggested that BMI is a poor predictor of sarcopenia, particularly in the aging population, as loss of lean muscle mass can coincide with an increase in tissue adiposity, resulting in relatively maintained BMI with decreased fat-free muscle mass [23].

To our knowledge, no other published research has used the newly established CT cutoff values that were implemented in this study as defined by Derstine et al. [19]. These values must continue to be studied with respect to opportunistic CT examination for sarcopenia so that more robust data can be cataloged regarding the utility of these cutoff values. Further research will help identify whether there truly exists such a discrepancy in diagnostic rates of sarcopenia based on the different muscle metrics as identified in this present study.

A few limitations were present in this study. One of the main limitations of our research was that both non-contrast and contrast-enhanced CT images were used to evaluate SMA and SMRA. Of the 89 scans used in this study, 16 were non-contrast CT and 73 were contrast-enhanced CT. Given the large difference between the two sub-groups of contrast and non-contrast CT and the lack of randomization to either contrast or non-contrast CT, statistical analysis comparing the total group and the sub-group data was not obtained. Although the cutoff values obtained for SMRA were established on non-contrast CTs, [18,19] other studies include both contrast-enhanced and non-contrast images of study patients [27]. Ideally, CT imaging used for muscle measurements are obtained on non-contrast imaging at 120 kV [27]. However, Van der Werf et al. found that IV CT contrast only slightly influences SMA and SMRA values [20] and our study aimed to evaluate a broader population by inclusion of contrast CT studies. To try to reduce measurement error, our study used a standard imaging parameter and IV contrast and contrast dose [20]. Additionally, this study had the potential for recall bias as the questionnaire for calculating MET-hours relied on patient recall of their level of exercise over the preceding 10 years. Finally, this study did not account for patient comorbidities such as presence of hip prosthesis, history of lumbar spinal fractures, or other patient factors that could potentially influence muscle mass or fat content at the L3 vertebral level. Due to the small participant sample size, the utility of statistical analysis including these variables seemed limited.

Given the plethora of metrics used to identify sarcopenia, we must further define which of those metrics is most strongly correlated with the adverse outcomes associated with sarcopenia. As sarcopenia is defined by both a deficit in muscle strength and either muscle quality or muscle quantity, it is important to identify potential deficits in the utilization of either quality or quantity metrics. It is clear that isolated use of either muscle quantity or quality metrics or inconsistent cutoff values results in substantial variability of identified prevalence of sarcopenia within a given population. A large difference in prevalence of sarcopenia existed within this sample population when diagnosing sarcopenia based on the different muscle metric cutoff values depending on whether SMA, SMI, or SMRA were used.

Opportunistic CT imaging of the abdomen and pelvis is the most promising imaging modality to provide the needed information in an automated and opportunistic fashion that is beneficial to patient care and health care costs. Additional studies using the cutoffs implemented in this study for SMA, SMI, and SMRA are needed to further characterize their diagnostic significance as it relates to the increased morbidity and mortality of sarcopenia so that opportunistic CT evaluation may be optimized. This will allow for better identification of the appropriate metrics to accurately identify patients at greatest risk for adverse outcomes associated with sarcopenia. Furthermore, if the most highly associated metric can be identified, work can be done to formulate interventions that specifically target that metric.

## 5 Conclusion

Exercise was found to have a positive association with SMRA, though this association was no longer significant when adjusting for the variables of BMI and cancer diagnosis. This finding may serve to signal an underlying significant positive association that requires a higher-powered multi-center study to identify the true association between these two variables. The positive association between SMRA and exercise is further reinforced by the significant lower odds of being diagnosed with sarcopenia using the SMRA threshold. This odds ratio suggested that the odds of identifying sarcopenia based on SMRA cutoffs decreased as participant exercise levels increased. This data suggests that SMRA may be the most useful metric to track when characterizing muscle metric response to exercise interventions. Although further research is needed to evaluate SMA, SMI, and SMRA, we believe that this association may indicate that SMRA may be a more relevant muscle metric than SMA or SMI, as it relates to muscle function for the preventative and possible rehabilitation of patents with sarcopenia.

## Human and animal rights

The authors declare that the work described has not involved experimentation on humans or animals.

## Informed consent and patient details

The authors declare that this report does not contain any personal information that could lead to the identification of the patient(s) and/or volunteers.

## Funding

This work did not receive any grant from funding agencies in the public, commercial, or not-for-profit sectors.

## CRediT authorship contribution statement

**Robert Janiszewski:** Data curation, Formal analysis, Project administration, Writing – original draft, Writing – review & editing. **Nathan Law:** Data curation. **Ryan Walters:** Formal analysis, Writing – review & editing. **Tami DenOtter:** Conceptualization, Data curation, Formal analysis, Funding acquisition, Investigation, Methodology, Project administration, Resources, Software, Supervision, Validation, Visualization, Writing – original draft, Writing – review & editing.

## Declaration of Competing Interest

The authors declare that they have no known competing financial or personal relationships that could be viewed as influencing the work reported in this paper.
